# Silent Myocardial Ischemia in CKD Stage 3-5: Prevalence and Predictors

**DOI:** 10.7759/cureus.92107

**Published:** 2025-09-11

**Authors:** Warda Batool Ali, Talha Tariq, Aneeqa Raashid Sidhu, Seher Anan, Summan Jannat, Muhammad Tayyab, Mehjabeen Ahmad, Umer Mushtaq

**Affiliations:** 1 Acute Medicine, Nottingham University Hospitals NHS Trust, Nottingham, GBR; 2 Internal Medicine, Rahim Yar Khan (RYK) Teaching Hospital, Rahim Yar Khan, PAK; 3 Medicine and Surgery, Bashir Begum Hospital, Burewala, PAK; 4 Emergency Medicine, AdLife Hospital, Al Amarat, OMN; 5 General Medicine, York and Scarborough Teaching Hospitals NHS Foundation Trust, York, GBR; 6 Cardiology, Azra Naheed Medical College, Lahore, PAK; 7 Internal Medicine, Nishtar Medical University and Hospital, Multan, PAK; 8 General Medicine, Jinnah Hospital, Lahore, PAK

**Keywords:** cardiac ischemia, chronic kidney disease (ckd), high bmi, silent myocardial ischemia, ventricular hypertrophy

## Abstract

Background: Silent myocardial ischemia (SMI) is a major but often underdiagnosed cardiovascular complication in patients with chronic kidney disease (CKD), particularly in advanced stages.

Objective: This study aims to determine the prevalence of silent myocardial ischemia and identify its clinical and biochemical predictors among patients with CKD stage 3-5.

Methods: This was a cross-sectional analytical study conducted at Allama Iqbal Medical College, Lahore, Pakistan, from January 2024 to December 2024. Data were collected using a structured questionnaire specifically designed for this study. Demographic information such as age, gender, and body mass index (BMI) was recorded for each participant.

Results: The prevalence of silent myocardial ischemia was found to be 37.4% (n = 89) in the study population. Patients with SMI were significantly older (mean age: 61.2 ± 10.5 years) compared to those without SMI (mean age: 57.1 ± 11.3 years) (p = 0.01). Diabetes mellitus was present in 82% of patients with SMI compared to 57.3% of those without (p < 0.001). Left ventricular hypertrophy (LVH) was observed in 55% of patients with SMI versus 31.4% of patients without SMI (p = 0.002). Elevated parathyroid hormone levels (>150 pg/mL) were seen in 73% of patients with SMI compared to 61% of patients without SMI (p = 0.047). Logistic regression analysis identified age > 60 years (adjusted odds ratio (AOR): 2.1; 95% confidence interval (CI): 1.2-3.5), diabetes mellitus (AOR: 2.9; 95% CI: 1.6-5.3), left ventricular hypertrophy (AOR: 2.4; 95% CI: 1.3-4.3), and elevated parathyroid hormone (PTH) levels (AOR: 1.8; 95% CI: 1.0-3.2) as independent predictors of SMI.

Conclusion: Silent myocardial ischemia is common in patients with CKD stage 3-5 and is independently associated with age, diabetes mellitus, left ventricular hypertrophy, and elevated PTH levels. Routine screening for SMI in high-risk patients with CKD is warranted to enable early detection and prevention of major cardiovascular events. Future longitudinal studies are recommended to assess the long-term impact of such screening and intervention strategies.

## Introduction

Chronic kidney disease (CKD) is identified as one of the most rapidly increasing global public health problems, and its estimation shows the rates of CKD affecting more than 10% of adults around the world [1]. There is a high risk of cardiovascular complications with the progression of CKD, through advanced CKD stages, which is pointed out by moderate to severe decreases in glomerular filtration rate (GFR). There is a range of complications, among which the problem of silent myocardial ischemia (SMI) is critical but largely ignored. SMI is also an objective finding of myocardial ischemia revealed by diagnostic testing in the absence of angina and other manifestations of ischemia [2]. The asymptomatic nature of silent myocardial ischemia is particularly dangerous because it delays timely preventive interventions, thereby directly contributing to the elevated cardiovascular morbidity and mortality observed in patients with CKD. The underlying pathophysiology that connects CKD and a high predisposition to SMI is multiple and complex. There is a synergistic effect of traditional and non-traditional risk factors of cardiovascular diseases in patients with CKD stage 3-5; traditional risk factors include hypertension, dyslipidemia, and diabetes mellitus, whereas non-traditional risk factors include endothelial dysfunction, vascular calcification, chronic inflammation, oxidative stress, and anemia [3,4]. CKD also favors the deposition of calcium in the arteries of the heart due to uremic toxins and mineral bone disorder, in addition to blunting the pain typical of myocardial ischemia due to left ventricular hypertrophy (LVH) and autonomic dysfunction that render the said phenomenon clinically silent in more than half the number of instances [5].

Varying degrees of prevalence of SMI among patients with CKD have been reported in the literature, and the prevalence might range depending on the study population, diagnostic criteria, and methodology utilized, between around 20% and more than 50% [6,7]. An example is stress myocardial perfusion imaging or coronary angiography studies. Studies usually report an increased prevalence as compared to those studies that use only standard electrocardiography. These fluctuations illustrate the necessity of population-specific studies, especially in areas where CKD has different epidemiology and resource distribution for healthcare.

Identifying the predictors of SMI in patients with CKD stage 3 or 5 is critical to the development of effective screening and prevention strategies. Among the predictors most frequently reported are old age and male gender, and these may relate to overall cardiovascular risks [8]. Another strong predictor is diabetes mellitus, which has a direct impact on the macrovascular and microvascular pathology of the coronaries. A long history of hypertension, anemia, a lack of calcium-phosphorus balance, proteinuria, and some echocardiographic signs of left ventricular hypertrophy are also important predictors [9,10]. Biochemical indicators of silent ischemia linked to CKD cohorts include increased high-sensitivity C-reactive protein (hs-CRP), hyperparathyroidism, and fibroblast growth factor 23 (FGF23). Notwithstanding the clinical significance of SMI, it may be under-recognized in clinical nephrology practice, primarily in the case of low- and middle-income countries where cardiovascular screening resources might be scarce. The general absence of screening for SMI is augmented by the fact that there are no defined guidelines for asymptomatic patients with CKD in particular. Whereas certain nephrology societies encourage cardiovascular risk stratification in patients with advanced CKD, there are great disparities in practice [11]. Non-invasive tests such as stress echocardiography, single-photon emission computed tomography (SPECT), cardiac magnetic resonance imaging (MRI), and ambulatory electrocardiogram (ECG) are useful for detecting silent myocardial ischemia but remain limited by cost, comorbidities, and accessibility [12]. SMI in CKD goes undiagnosed, but its clinical outcomes are serious. Several studies have indicated that SMI is linked with a higher risk of major adverse cardiovascular events (MACEs), such as myocardial infarction, heart failure, and sudden cardiac death [13]. In addition, silent ischemia is one of the factors in the high all-cause and cardiovascular mortality of patients with CKD stage 3-5 that have no dependence on other risk factors.

The basic aim of this study was to determine the prevalence of silent myocardial ischemia and identify its clinical and biochemical predictors in patients with CKD stage 3-5.

## Materials and methods

This was a cross-sectional analytical study conducted at Allama Iqbal Medical College, Lahore, Pakistan, from January 2024 to December 2024. This study was approved by the Institutional Review Board (IRB) of Allama Iqbal Medical College, Lahore, with approval number 246/ERB.

Sample size and sampling technique

A total of 238 adult patients diagnosed with CKD stage 3, 4, or 5 were enrolled in the study. The sample size was determined based on previous prevalence rates of SMI in CKD populations, considering a 95% confidence level and 5% margin of error [14]. Participants were recruited using a non-probability consecutive sampling technique from the nephrology outpatient clinics and inpatient wards of Allama Iqbal Medical College-affiliated hospitals during the study period. All eligible individuals with CKD stage 3-5, as determined by estimated glomerular filtration rate (eGFR) using the CKD-EPI formula, were approached for participation.

Inclusion and exclusion criteria

The inclusion criteria for this study comprised patients aged 18 years and above with confirmed CKD stage 3-5, as classified according to estimated glomerular filtration rate (eGFR) using the CKD-EPI formula. All included patients were clinically stable and willing to undergo cardiovascular assessment. Exclusion criteria involved patients with a documented history of symptomatic coronary artery disease, prior myocardial infarction, coronary revascularization, or heart failure. Patients undergoing dialysis and those with active infections, malignancies, or severe anemia (defined as hemoglobin levels below 7 g/dL) were excluded from the study to maintain a homogeneous sample reflective of stable CKD stage 3-5 without overt cardiac symptoms.

Data collection procedure

Data were collected using a structured proforma specifically designed for this study. Demographic information such as age, gender, and body mass index (BMI) was recorded for each participant. Clinical data, including CKD stage, duration of kidney disease, presence of comorbid conditions such as diabetes mellitus and hypertension, smoking status, and lipid profile parameters, were noted. Laboratory assessments included hemoglobin concentration, serum creatinine levels, calculated eGFR, serum calcium, phosphorus, and parathyroid hormone (PTH) levels, as these variables are relevant both to CKD progression and cardiovascular risk assessment.

Assessment of silent myocardial ischemia

All enrolled participants underwent a structured cardiovascular evaluation to detect silent myocardial ischemia. A resting 12-lead electrocardiography (ECG) was performed initially to identify ischemic changes such as ST-segment depression or T-wave inversions in the absence of chest pain or equivalent symptoms. Asymptomatic ischemia was defined as a horizontal or downsloping ST-segment depression of 12 mm or more at 80 ms after the J point or T-wave inversion in two or more continuous leads. Patients capable of exercising underwent a treadmill exercise test by the Bruce protocol, where positivity was established as 1 mm or more horizontal or downsloping ST depression, failure to reach an age-predicted maximum heart rate, or arrhythmias manifested by exercise. Patients who could not be tested with an exercise test received a myocardial perfusion imaging (MPI) test, in which an abnormality was considered a perfusion defect that involved at least 10% of left ventricular myocardium or a fixed/reversible defect that involved at least two adjacent myocardial segments. All patients also had transthoracic echocardiography to assess left ventricular hypertrophy (abnormal left ventricular (LV) mass index; 115 g/m² in men and 95 g/m² in women), abnormal regional wall motion, and left ventricular ejection fraction (LVEF < 50% is considered abnormal). Any ischemic abnormalities in each of these modalities in the absence of anginal symptoms or equivalents were termed silent myocardial ischemia.

Statistical analysis

The collected data were analyzed using SPSS version 29.0 (IBM Corp., Armonk, NY). Quantitative variables such as age, BMI, serum creatinine, and lipid profile levels were presented as mean ± standard deviation (SD), while categorical variables such as gender, CKD stage, presence of diabetes mellitus, and occurrence of silent myocardial ischemia were summarized using frequencies and percentages. To compare categorical variables between patients with and without silent myocardial ischemia, the chi-square test was applied. A p-value less than 0.05 was considered statistically significant throughout the analysis.

## Results

Data were collected from 238 patients with CKD stage 3-5. The mean age was 58.7 ± 11.2 years, with nearly half (114 (47.9%)) aged over 60. Male patients made up 142 (59.7%) of the sample. Diabetes was present in 157 (66%) and hypertension in 194 (81.5%) overall, both more frequent in the SMI group (82% and 86.5%, respectively) compared to non-SMI. A sedentary lifestyle was common, reported in 172 (72.3%) patients. The SMI group had slightly higher mean BMI (27.1 kg/m²) and age (61.2 years) compared to non-SMI patients, suggesting that older, more metabolically burdened patients were more prone to silent myocardial ischemia (Table 1).

**Table 1:** Demographic and clinical characteristics of patients with CKD stage 3-5 (N = 238) SMI: silent myocardial ischemia, SD: standard deviation, BMI: body mass index

Characteristic	Total (N = 238)	SMI group (n = 89)	Non-SMI group (n = 149)
Age (years), mean ± SD	58.7 ± 11.2	61.2 ± 10.5	57.1 ± 11.3
Age > 60 years	114 (47.9%)	55 (61.8%)	59 (39.6%)
Male gender	142 (59.7%)	54 (60.7%)	88 (59.1%)
Diabetes mellitus	157 (66%)	73 (82%)	84 (57.3%)
Hypertension	194 (81.5%)	77 (86.5%)	117 (78.5%)
Smoking history	51 (21.4%)	21 (23.6%)	30 (20.1%)
Sedentary lifestyle	172 (72.3%)	68 (76.4%)	104 (69.8%)
BMI (kg/m²), mean ± SD	26.9 ± 4.5	27.1 ± 4.3	26.8 ± 4.6

Biochemical comparison showed that mean hemoglobin, serum creatinine, and eGFR levels were similar between SMI and non-SMI groups, with no statistically significant difference (p > 0.05 for all). However, hyperphosphatemia was slightly more prevalent in the SMI group (59.5% versus 51.7%), although not statistically significant (p = 0.27). Elevated PTH was notably higher in the SMI group (73% versus 61%) with a borderline p-value of 0.047. Left ventricular hypertrophy was significantly more frequent in patients with SMI (55% versus 31.4%; p = 0.002), highlighting a strong cardiac remodeling association in these patients (Table 2).

**Table 2:** Biochemical parameters in patients with and without silent myocardial ischemia SMI: silent myocardial ischemia, SD: standard deviation, eGFR: estimated glomerular filtration rate, PTH: parathyroid hormone, LV: left ventricular

Parameter	SMI group (n = 89)	Non-SMI group (n = 149)	Test statistic	p-value
Hemoglobin (g/dL), mean ± SD	9.6 ± 1.8	9.9 ± 1.6	t = 1.26	0.20
Serum creatinine (mg/dL), mean ± SD	5.4 ± 2.0	5.2 ± 2.2	t = 0.74	0.46
eGFR (mL/min/1.73 m²), mean ± SD	21.9 ± 8.6	23.1 ± 9.1	t = 1.04	0.30
Hyperphosphatemia (>4.5 mg/dL)	53 (59.5%)	77 (51.7%)	χ² = 1.23	0.27
Elevated PTH (>150 pg/mL)	65 (73%)	91 (61%)	χ² = 3.97	0.047
LV hypertrophy on echo	49 (55%)	47 (31.4%)	χ² = 11.07	0.002

**Figure 1:**
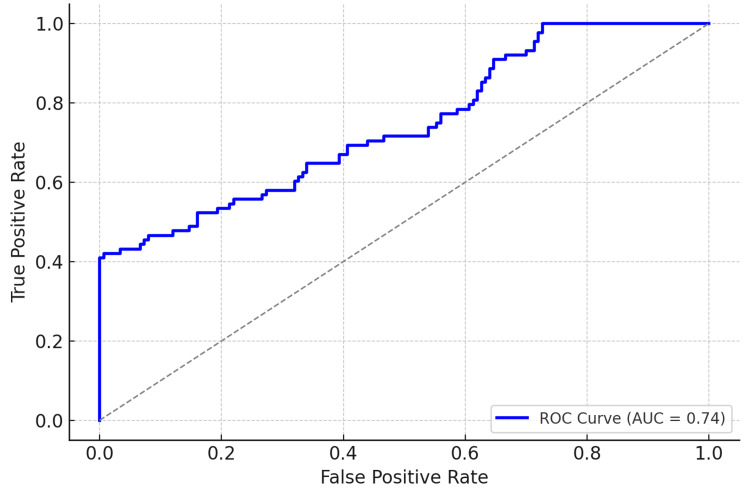
ROC curve for the SMI prediction model, demonstrating moderate discriminatory ability with an AUC of 0.74 ROC: receiver operating characteristic, SMI: silent myocardial ischemia, AUC: area under the curve

Out of all 238 patients with CKD stage 3-5, silent myocardial ischemia was detected in 89 individuals, representing 37.4% of the cohort. Within this subgroup, positive stress test results were seen in 41 (46.1%), while myocardial perfusion imaging revealed perfusion defects in 28 (31.5%). Additionally, echocardiography abnormalities suggestive of ischemia were identified in 20 (22.4%) of SMI cases (Table 3, Figure 1).

**Table 3:** Prevalence and detection of SMI among patients with CKD stage 3-5 (N = 238) SMI: silent myocardial ischemia, CKD: chronic kidney disease, MPI: myocardial perfusion imaging

Parameter	Frequency (number)	Percentage (%)
Silent myocardial ischemia (total)	89	37.4%
Positive stress test	41	46.1% (of SMI)
Perfusion defects on MPI	28	31.5% (of SMI)
Echo abnormalities suggestive of ischemia	20	22.4% (of SMI)

Logistic regression revealed that age over 60 years independently predicted silent myocardial ischemia with an adjusted odds ratio (AOR) of 2.1 (p = 0.004). Diabetes mellitus emerged as the strongest predictor (AOR = 2.9; p < 0.001), followed by left ventricular hypertrophy (AOR = 2.4; p = 0.002). Elevated PTH also contributed as a significant predictor with an AOR of 1.8 (p = 0.047) (Table 4).

**Table 4:** Logistic regression analysis for predictors of silent myocardial ischemia AOR: adjusted odds ratio, PTH: parathyroid hormone

Predictor	AOR	95% confidence interval	p-value
Age > 60 years	2.1	1.2-3.5	0.004
Diabetes mellitus	2.9	1.6-5.3	<0.001
Left ventricular hypertrophy	2.4	1.3-4.3	0.002
Elevated PTH (>150 pg/mL)	1.8	1.0-3.2	0.047

## Discussion

The prevalence and clinical predictors of silent myocardial ischemia (SMI) in patients with chronic kidney without dialysis (CKD) stage 3-5 were discussed with 238 being the number of participants. The findings indicated how a significant proportion as large as 37.4% experience SMI, which testifies to the existence of unnoticed cardiovascular risk in these groups of patients. This observation agrees with the available literature that postulates rates of SMI in patients with CKD as between 20% and 50%, although specific numbers tend to differ according to the method of diagnosis and study location [14]. Our findings of the high prevalence indicate the evidence that the prevalence of SMI is a severe but overlooked issue of cardiovascular disease in CKD. Of possible reasons, the occurrence of autonomic neuropathy and deficits in pain perception of CKD, particularly in the diabetic subtype, dulls the endocardial process so that the classic anginal subtypes are presented as late-stage non-specific manifestations of a full-blown myocardial infarction or sudden cardiac death [15].

One of the identified factors that is linked to SMI is age of more than 60 years, and it turned out to be a significant indicator. Age is a proven risk factor for cardiovascular diseases, and there is also an increase in atherosclerosis and vascular stiffness with older age and ischemic potential. Our results support the overall knowledge fact that increasing age on its own predisposes symptomatic and silent acute coronary events in people with CKD [16].

The strongest independent risk factor was diabetes, present in 66% of the total number of participants in the present study, with an adjusted odds ratio (AOR) of 2.9. The observed relationship is also in line with other literature where diabetic nephropathy is presumed to cause a greater than normal share of cardiovascular morbidity in CKD. Diabetes worsens endothelial dysfunction, facilitates coronary artery calcification, and leads to left ventricular hypertrophy and heart failure, which all predispose to the occurrence of unnoticed ischemia [17,18].

The total cohort and 55% of the patients with SMI were demonstrated to have left ventricular hypertrophy (LVH). The criterion LVH indicates chronic volume and pressure overload associated with states of hypertension, anemia, and arteriovenous fistulas related to CKD. It not only heightens myocardial oxygen expenditure but also facilitates diastolic dysfunction, both of which predispose to ischemia. These findings are consistent with results of the Chronic Renal Insufficiency Cohort (CRIC) study and other global registries in which LVH is an important mediator of cardiovascular risk [19].

The other interesting finding was that an increase in parathyroid hormone (PTH) (150 pg/mL and above) was associated with SMI. Secondary hyperparathyroidism, which commonly develops in patients with chronic kidney disease (CKD stage 3-5), contributes to vascular calcification, atherosclerosis, and myocardial fibrosis, ultimately increasing the overall ischemic burden. Although this relationship has been reported anecdotally in smaller observational studies, our present cross-sectional study develops additional statistical evidence toward the hypothesis that disturbances in mineral metabolism are not only a cause of structural vascular changes but also of functional cardiac events such as SMI [20].

Interestingly, our study did not indicate a statistically significant relationship between SMI and gender, smoking status, and BMI. It differs from the non-CKD population, where these have otherwise been found to be traditional risk factors. An alternative way would be the fact that CKD-related non-traditional risk factors, i.e., anemia, oxidative stress, chronic inflammation, and uremic toxin accumulation, would have a stronger effect on cardiovascular outcomes compared to the classic risk profiles [21]. These findings are medically important. Silent myocardial ischemia in patients with stage 3-5 CKD is not a rare phenomenon; quite on the contrary, it is rather frequent and implies definite pathogens. The identification of risk factors such as age, diabetes mellitus, LVH, and high PTH levels will help clinicians have practical measures of risk stratification. This implies that there needs to be specific cardiovascular screening instruments among high-risk patients with CKD to screen them specifically, since this may not be possible in resource-limited healthcare facilities where all patients with CKD require regular cardiac imaging tests. Considering our findings, the inclusion of non-invasive cardiac assessment in the form of treadmill exercise test, myocardium perfusion imaging, or stress echocardiogram in the protocol of patients with diabetes and LVH with CKD should be considered even when they are asymptomatic, being part of the older CKD population. An active diagnosis and treatment may resolve the frequency of major adverse cardiovascular events (MACEs), which are the leading cause of death in the CKD population worldwide [22].

Limitations

This study has several limitations that must be acknowledged. First, its cross-sectional design limits the ability to establish causal relationships between the identified predictors and the occurrence of silent myocardial ischemia (SMI). Second, the sample was drawn from a single center, which may restrict the generalizability of the findings to broader CKD populations with different demographic or clinical profiles. Future research should validate these findings in larger, multicenter cohorts to improve generalizability across diverse CKD populations. Longitudinal studies are needed to determine the prognostic impact of silent myocardial ischemia on major adverse cardiovascular events in CKD.

## Conclusions

It is concluded that silent myocardial ischemia is a significant yet under-recognized concern in patients with moderate to advanced chronic kidney disease. The observed association with both traditional risk factors and CKD-specific complications underscores the need for proactive cardiovascular risk assessment in this population. Integrating routine screening for silent ischemia into standard nephrology care may enable earlier intervention, potentially reducing cardiovascular morbidity and improving long-term outcomes for these patients.
